# Older adults respond better to psychological therapy than working-age adults: evidence from a large sample of mental health service attendees.

**DOI:** 10.1016/j.jad.2021.06.084

**Published:** 2021-11-01

**Authors:** Rob Saunders, Joshua E.J. Buckman, Joshua Stott, Judy Leibowitz, Elisa Aguirre, Amber John, Glyn Lewis, John Cape, Stephen Pilling, NCEL network

**Affiliations:** a Centre for Outcomes Research and Effectiveness, Research Department of Clinical, Educational and Health Psychology, University College London, Gower Street, London, UK; b iCope – Camden and Islington Psychological Therapies Services, Camden & Islington NHS Foundation Trust, London, UK; c ADAPT lab, Research Department of Clinical, Educational and Health Psychology, University College London, Gower Street, London, UK; d North East London NHS Foundation Trust, London, UK; e Division of Psychiatry, University College London, London, W1T 7NF, UK; f Camden & Islington NHS Foundation Trust, St Pancras Hospital, 4 St Pancras Way, London, UK

**Keywords:** Depressive disorder, Anxiety disorders, Psychological therapy, Psychotherapy outcome research, Geriatric psychiatry, Community mental health services

## Abstract

**Background:**

Older adults commonly experience depression and anxiety, yet are under-represented in psychological treatment services. There is uncertainty about the outcomes from psychological therapies for older adults relative to working-age adults. This study explored: pre-treatment differences between older and working-age patients with depression or anxiety disorders; whether outcomes from psychological therapy differ between groups controlling for pre-treatment clinical severity, functioning, and socio-demographics; and whether the impact of a long-term health condition (LTC) on outcome differs by age.

**Methods:**

Data on >100,000 patients treated with psychological therapies in eight Improving Access to Psychological Therapies services were analyzed. We compared pre-treatment characteristics and therapy outcomes for older (≥65 years) and working-age (18-64 years) patients, and investigated associations between age and outcomes.

**Results:**

Older adults had less severe clinical presentations pre-treatment. In adjusted models older adults were more likely to reliably recover (OR=1.33(95%CI=1.24-1.43)), reliably improve (OR=1.34(95%CI =1.24-1.45)), and attrition was less likely (OR=0.48(95%CI =0.43-0.53)). Effects were more pronounced in patients with anxiety disorders compared to depression. Having an LTC was associated with a much lower likelihood of reliable recovery for working-age patients but had only a modest effect for older adults.

**Limitations:**

There are potential selection biases affecting the characteristics of older people attending these services. Residual confounding cannot be ruled out due to limits on data available.

**Conclusions:**

Older adults experienced better outcomes from psychological treatments than working-age adults. Given the deleterious effects if mental health conditions go untreated, increasing access to psychological therapies for older people should be an international priority.

## Highlights

• There is a belief that older people do not benefit from psychological interventions.
• Older people had less severe symptoms pre-treatment compared to working-age people.
• Clinical improvement was more likely and attrition less likely among older patients.
• Effects held when adjusting for all baseline characteristics and treatment factors.
• Age-group effects were largest for those with LTCs or anxiety disorders.

## 1 Introduction

A quarter of older adults (over 65s) have a common mental disorder (CMD) such as depression or an anxiety disorder (Evans and Mottram, 2000; Gowling et al., 2016). CMDs in older adults are associated with negative outcomes including increased risk of cognitive impairment and dementia (Byers and Yaffe, 2011; Kazmi et al., 2021) and earlier mortality (Saz and Dewey, 2001). Psychological therapies such as cognitive behaviour therapy (CBT) are proven to be effective (NICE, 2011), there is a patient preference for psychological therapies over antidepressant medications (Cuijpers et al., 2020; McHugh et al., 2013), and there are fewer adverse side-effects (Carvalho et al., 2016). However, CMDs are more likely to be treated with psychotropic medication (including antidepressants) than psychological therapies in many parts of the world (Maust et al., 2017; Tamblyn et al., 2019), and this is more pronounced among older adults, particularly in the USA (Sanglier et al., 2011). Consequently, older adults are currently under-represented in services offering psychological treatments for CMDs (Clark, 2018). This may be due to beliefs by clinicians and referrers that psychological interventions for CMDs are less effective in this patient group (Mental Health Taskforce, 2016), but also from older adults who consider themselves as less likely to benefit from psychological treatments (Laidlaw et al., 2003).

Meta-analytic reviews suggest that interventions for depression are equally effective for older and working-age adults (Cuijpers et al., 2018; Haigh et al., 2018), but that older adults may be less likely to benefit from psychological treatments for anxiety disorders (Gould et al., 2012). However, past reviews have typically compared effect sizes of trials of older adults with the effect sizes of trials of working-age adults, without being able to assess the impact of age on outcomes or make adjustments for pre-treatment differences in the presentations of older and working-age patients, using individual patient data (Cuijpers et al., 2018; Haigh et al., 2018). Reviews that have used individual patient data have been restricted by the number of older adults in their samples, and the lack of data on outcomes from psychological therapies (Buckman et al., 2021a). As such, the evidence base on the effectiveness of routine psychological treatments for older adults is limited, and due to the small sample size of most psychological treatment trials and limited power in study-level meta-analyses, estimates of such effects have been imprecise (Buckman et al., 2021b). Further, as the setting in which treatment was conducted has been unclear or allowed to vary in past studies and reviews in order to maximise sample size (Cuijpers et al., 2018; Haigh et al., 2018), the generalizability of reported associations to settings in which the majority of depressed or anxious adults might be treated with psychological therapies is dubitable (Buckman et al., 2021a). If there are differences in the outcomes of psychological therapies for older and working-age adults, one potential reason for this could be the prevalence of long-term health conditions (LTCs) among older adults and the impact of such conditions on therapy outcomes (Callahan, 2001; Laidlaw et al., 2003; Unützer et al., 2000). LTCs such as arthritis, diabetes, hypertension, cardiac problems, and pulmonary diseases are more common among older adults, and are all associated with functional impairments. LTCs and common mental disorders are highly comorbid (Djernes, 2006) and this comorbidity is associated with poorer prognoses both for the LTC and the mental health condition (Callahan, 2001; Unützer et al., 2000). In particular, LTCs and associated impairments may negatively affect engagement with, and the outcome of, psychological therapies for depression and anxiety disorders (Amati et al., 2018; Catarino et al., 2018). Symptoms of anxiety or depression may pre-date the onset of comorbid LTCs (Cosci et al., 2015) or arise after the onset of a LTC, but there is some evidence that psychological therapies can be effective irrespective of the chronological order of mental and physical disorders (Fava, 2016; Fava et al., 2016).

Given the prevalence of LTCs and their association with older age, we might expect that in routine mental health services older adults would be more likely to disengage from psychological therapy and have worse treatment outcomes than working-age adults. That some older people (Laidlaw et al., 2003), as well as some clinicians (Mental Health Taskforce, 2016), do not believe they will be able to benefit is likely stimulated by earlier suggestions of limited effectiveness of psychological treatments for older adults (Chand and Grossberg, 2013; Stanley et al., 2009). However, evaluations of psychological treatment services as part of the Improving Access to Psychological Therapies (IAPT) programme in England consistently show that over 65s have significantly higher recovery rates (64.4%) following psychological therapy than working-age adults (50.2%) (NHS Digital, 2020). As these evaluations use aggregate data from each service, they are unable to adjust for any pre-treatment differences in the clinical presentations of older and working-age adults, which might explain some or all of the difference in their outcomes. It may be the case that older people with more severe problems or with comorbid LTCs are not referred, do not want to attend, or are screened out of treatment in such services, they may have less severe symptomatology, attend more sessions or have different waiting times, all of which have previously been found to be associated with better treatment outcomes (Clark et al., 2018; Saunders et al., 2019). Given that the number of over 65’s worldwide is projected to increase by approximately 60% by 2030 (He et al., 2016), there is a pressing need to understand whether routinely available psychological therapy is effective for older adults with CMDs to meet the needs of this patient group, something which is now an international priority (World Health Organization, 2016). Therefore, understanding potential differences among working-age and older patients attending psychological treatment services and whether any pre-treatment differences influence treatment outcomes, could be of great importance.

We aimed to investigate the differences in the pre-treatment characteristics and outcomes for older and working-age patients treated with psychological therapies for anxiety or depression. In addition, we aimed to investigate outcomes separately for those with a primary diagnosis of depression and those with an anxiety disorder, and to compare outcomes for those with and without a comorbid long-term physical health condition.

## 2 Methods

### 2.1 Services

Data were provided by eight Improving Access to Psychological Therapies (IAPT) services based in London, England. These National Health Service (NHS) primary care and community-based mental health teams deliver evidence-based psychological therapies for depression and anxiety disorders. They offer a range of low-intensity (LI) treatments such as guided self-help, and formal high intensity (HI) psychological interventions such as cognitive behaviour therapy or counselling, all delivered utilising a stepped-care model in line with national guidelines and evidence-based practice (see (Clark, 2018) for more details). All services were members of the North and Central East London IAPT Service Improvement and Research Network (NCEL IAPT SIRN) (Saunders et al., 2020).

### 2.2 Participants

A retrospective dataset was formed from all 132,403 patients referred to the services between August 2008 and May 2019 who received two or more sessions of treatment, the minimum requirement for an episode of care as defined in national evaluations of IAPT services (NHS Digital, 2019). Those aged 65 years or older were defined as older adults, those under 65 were considered working-age adults, this is also in line with national evaluations of these services. Patients were included in the analysis if they: were 18 years old or above at referral; had completed their episode of treatment (i.e. were not still receiving treatment), and were above the clinical cut-off for ‘caseness’ on any of the depression or anxiety symptom measures used in the services at assessment (see Measures section below). From the available dataset, 2,307 patients were under 18 years, 5,286 were still in treatment, and 12,983 did not score above the cut-off for caseness, and were excluded. Patients were also excluded from these analyses if they were missing data related to age (n=14) or did not provide a post-baseline set of outcome measures as they did not have data with which to calculate the study outcomes (n=5,010). Furthermore, patients who had a primary presenting problem for which there is no recommended evidence-based treatments in IAPT services (Clark, 2018) such as schizophrenia, bipolar disorder or alcohol dependency were also excluded (n=6,624). This resulted in a sample of 100,179 patients available for analyses. A full patient flow diagram is presented in Appendix A. NHS ethical approval was not required for this study (confirmed by the Health Research Authority July 2020, reference number 81/81). The data were provided by the IAPT services for evaluation as part of a wider service improvement project conducted in accordance with the procedures of the host institution and the NHS Trusts which operate the IAPT services (project reference: 00519-IAPT).

### 2.3 Measures

The services routinely collect outcome measures of depression and anxiety symptoms at each clinical contact. Table 1 presents these self-report measures and additional data items that were included in the current analyses.

**Table 1:** Available data and measures.

Data Item	Questionnaire	Information on measurement
Depression	Patient Health Questionnaire 9-item (PHQ-9) (Kroenke et al., 2001)	To measure symptoms of depression, scores of 10 or above indicate clinical caseness for depression, and a reduction of 6 or more points is used to indicate reliable improvement (NHS Digital, 2016).
Anxiety	The Generalized Anxiety Disorder Scale 7-item version (GAD7) (Spitzer et al., 2006)	To assess generalized anxiety symptoms, a cut-off of 8 or higher is used for caseness and 4 or more for reliable improvement. Alternative “anxiety disorder specific measures” (ADSMs) are used when specific anxiety disorders are identified as the “problem descriptor” (Clark, 2018), for example the Social Phobia Inventory (Connor et al., 2000) for use when social anxiety disorder is identified. When present, these ADSMs are used to calculate outcomes instead of the GAD-7. The full list of ADSMs alongside the service thresholds for caseness and reliable change is presented in Appendix B and further details are available in the annual reports on the UK IAPT programme (NHS, 2018).
Personal functioning	The Work and Social Adjustment Scale (WSAS) (Mundt et al., 2002)	Measures personal functioning in relation to: ‘ability to work’, ‘home management’, ‘social activities’, ‘private leisure activities’ and ‘close relationships’ (domain score range, 0-8). The WSAS item on the ‘ability to work’ is routinely recorded as “not applicable” for individuals not in employment, as was the case for 85% of older adults in the present study sample. As a result, this item was excluded from the analyses.
Phobic anxiety	The IAPT Phobia Scales (National IAPT Programme Team, 2011)	Consist of three questions each assessing the degree of avoidance of certain situations related to different types of phobic anxiety - agoraphobia, social phobia and specific phobias
Problem descriptor	N/A	The services collect data on each patient's problem descriptor (ICD-10 code), representing a probable or confirmed diagnosis, in order to match patients to evidence-based treatments. We categorised problem descriptors following conventions from previous studies that used similar data (Buckman et al., 2018): depression; mixed anxiety and depression; generalized anxiety disorder; obsessive compulsive disorder; post-traumatic stress; phobic anxiety and panic. In addition, bereavement was kept as a separate category due to its expected prevalence in the older adult population.
Demographics	N/A	Self-reported gender at point of referral, age, index of multiple deprivation (IMD) decile and ethnicity (based on UK census codes ‘White’, ‘Mixed’, ‘Asian’, ‘Black’, ‘Chinese’ and ‘other’) were available in the dataset.
Long-term health conditions	N/A	All patients are asked whether or not they have any long-term physical health condition (LTC). The type of condition was not available in the dataset.
Medication	N/A	At every clinical contact with their patients clinicians in the services routinely record whether their patients were prescribed psychotropic medication(s).
Treatment factors	N/A	The number of “Low Intensity” (LI) and “High Intensity” (HI) treatment sessions received; the number of days between referral to the service and assessment, and the number of days between the assessment and first treatment session. Days between referral and sessions, and days between assessment and first treatment session were winsorized at the top 99% due to a limited number of extreme values.

### 2.4 Outcomes

#### 2.4.1 Primary

Reliable Recovery: moving from ‘caseness’ before treatment on either the PHQ-9 or the GAD-7 (or an Anxiety Disorder Specific Measure (ADSM)) to below ‘caseness’ for both depression and anxiety at the last appointment, as well as reporting a reduction in symptom scores above the error of measurement for either measure (reliable improvement). Thresholds for caseness and improvement for the PHQ-9 and GAD-7 are presented in Table 1, and thresholds for ADSMs in Appendix B. This outcome metric is used by IAPT services as well as national evaluations of services (Clark et al., 2018; NHS Digital, 2019).

#### 2.4.2 Secondary

• Reliable Improvement: reporting a reduction in symptom scores above the error of measurement for either the PHQ-9 or GAD-7 (or other ADSM), or both.
• Reliable deterioration: a reliable increase above the error of measurement in symptoms on any symptom-based outcome measure.
• Attrition: whether or not an individual is reported to have dropped out during their episode of care after receiving three or more treatment sessions. This is a subjective judgement made by the clinician to denote the reason the episode of care finished. Patients with fewer than three treatment sessions were not included for this outcome nor were those that were referred on for further care after having three of more sessions (11.5% of the full dataset).

#### 2.4.3 Plan of analysis

This study comprised of the following analyses:

1) Comparing older and working-age adults on all available baseline characteristics. Independent samples t-tests were used to explore differences in means of continuous variables between groups, and chi-square tests were used for categorical variables.

2) Exploration of whether age group is associated with outcomes whilst controlling for available confounders such as baseline severity, number of treatment sessions and socio-demographics, in a series of regression analyses. Analyses were first performed using age as a continuous variable, as well as a set of age bands (18-24 years; 25-44 years; 45-64 years and 65+ years) to assess the potential linear impact of age on the primary outcome, before treating age group as a binary variable (18-64 and 65+ years). Logistic regression models were constructed to explore the associations between age group and each outcome after controlling for baseline confounders. Missing baseline data were imputed using multiple imputation with chained equations using “ICE” (Royston and White, 2011) in Stata (StataCorp, 2019). Imputation models included all variables listed in Table 1 and were run to give 50 imputed datasets. Sensitivity analyses were conducted with complete data only. Five models were built for each outcome:

- Model 1: The crude association between age group and outcome.

- Model 2: As in 1, additionally adjusted for the service, number of treatment sessions and wait times.

- Model 3: As in 2, additionally adjusted for baseline PHQ-9, and GAD-7 scores.

- Model 4: As in 3, additionally adjusted for the four WSAS items (excluding the ‘work’ item as this is coded “not applicable” for retired people), and three phobia scale items at baseline.

- Model 5: As in 4, additionally adjusted for gender, ethnicity, medication, deprivation (Indices of Multiple Deprivation rank: IMD), problem descriptor, and LTC status.

Multilevel logistic regression models with service included as a random effect were also run to explore the impact of service-level clustering on outcomes. If results from the two types of models differed considerably, the multilevel models would be retained and used as the primary means of modelling for all analyses.

3) Further sensitivity and subgroups analyses were then conducted. Firstly, propensity score matching (Austin, 2011) was performed to identify matched groups of older and working-age adults to explore whether the two age groups differ in outcomes when they are very similar on other key variables (for more detail on methods please see Appendix C). Logistic regression models were then built to explore the association between age group and each of the four outcomes in the matched sample. This analysis was conducted controlling for all confounders (i.e. replicating Model 5 described above) to provide a conservative estimate of the impact of age group on each outcome.

Subgroup analyses were then performed to explore differences in outcomes between older and working-age patients: 1) treated for depression compared to those treated for anxiety disorders, and 2) between patients with and without LTCs, within the age groups. Differences in the treatment pathways of older and working-age in the sample were also explored, these were categorised as: 1) only LI treatment; 2) only HI treatment; 3) Stepped down (from HI to LI) or 4) Stepped up (from LI to HI) during their treatment episode. The type of treatment recorded at the final completed treatment session was compared for older and working-age adults. This may not have been the only treatment type received during the episode of care, but this aligns with national reporting on treatments received in IAPT (Community and Mental Health team, 2016). Finally, the total number of treatment sessions was compared between older and working-age adults. Linear and Multinomial logistic regression models were constructed to assess whether being an older adult was associated with treatment pathway, last treatment type and differences in the total number of sessions received, controlling for variables listed in Model 5 above.

## 3 Results

### 3.1 Comparison of older and working-age adults

Of the 100,179 patients that met inclusion criteria 3849 (3.8%) were older adults (65 years of age at referral or older). Comparisons of baseline clinical and demographic information between older and working-age adults are presented in Table 2. Older adults had less severe symptomatology at initial assessment, reported less impact on personal functioning and fewer phobic symptoms than working-age adults. They were also more likely to identify as female, report an LTC and be from a white ethnic group. Older adults attended fewer HI treatment sessions on average than working-age adults, and did not wait as long to be seen for assessment or to start treatment as working-age patients did.

**Table 2:** Comparison of baseline descriptive statistics between working-age and older adults groups.

		18-64 years			65± years			Difference p-value
		n	mean	sd	n	mean	sd	
PHQ-9		96313	15.65	5.61	3848	13.93	5.53	<0.001
GAD-7		96295	14.22	4.41	3846	12.77	4.65	<0.001
WSAS-item 2		83201	3.76	2.44	3260	3.29	2.49	<0.001
WSAS-item 3		83189	4.57	2.44	3259	3.70	2.61	<0.001
WSAS-item 4		83174	3.89	2.57	3258	3.27	2.53	<0.001
WSAS-item 5		83169	4.22	2.45	3255	3.02	2.53	<0.001
Agoraphobia item		93412	2.90	2.79	3663	2.29	2.63	<0.001
Social phobia item		93456	3.37	2.62	3663	2.38	2.52	<0.001
Specific phobia item		93411	2.44	2.81	3662	2.19	2.80	<0.001
Number LI sessions		96330	2.85	2.81	3849	2.98	3.02	0.007
Number HI sessions		96330	4.79	5.51	3849	4.26	5.07	<0.001
Days - referral to assessment		96269	27.18	32.86	3841	26.21	30.27	0.051
Days - assessment to treatment		96329	72.18	79.54	3849	68.68	81.37	0.009

		18-64 years			65±			
		n	%		n	%		p-value

Gender	Male	31197	32.39%		1173	30.48%		0.033
	Female	64390	66.84%		2641	68.62%		
	Missing	743	0.77%		35	0.91%		
LTC Case	No	54052	56.11%		1187	30.84%		<0.001
	Yes	19942	20.70%		1799	46.74%		
	Missing	22336	23.19%		863	22.42%		
Ethnicity (ONS)	White	61586	63.93%		2932	76.18%		<0.001
	Mixed	5562	5.77%		56	1.45%		
	Asian	8568	8.89%		223	5.79%		
	Black	10690	11.10%		221	5.74%		
	Chinese	550	0.57%		10	0.26%		
	Other	3315	3.44%		101	2.62%		
	Missing	6059	6.29%		306	7.95%		
Psychotropic medication	Prescribed - not taking	7750	8.05%		314	8.16%		0.002
	Prescribed and taking	35598	36.95%		1474	38.30%		
	Not prescribed	45170	46.89%		1700	44.17%		
	Missing	7812	8.11%		361	9.38%		
Index of Multiple Deprivation (IMD) Decile	1	10805	11.22%		305	7.92%		<0.001
	2	28632	29.72%		870	22.60%		
	3	21079	21.88%		707	18.37%		
	4	10791	11.20%		420	10.91%		
	5	7459	7.74%		355	9.22%		
	6	5892	6.12%		284	7.38%		
	7	3396	3.53%		254	6.60%		
	8	3327	3.45%		287	7.46%		
	9	1246	1.29%		154	4.00%		<0.001
	10	364	0.38%		47	1.22%		
	Missing	3339	3.47%		166	4.31%		
Problem descriptor	Depression	34170	35.47%		1456	37.83%		
	MADD	7712	8.01%		248	6.44%		
	GAD	13299	13.81%		558	14.50%		
	OCD	1641	1.70%		29	0.75%		
	PTSD	3083	3.20%		52	1.35%		
	Phobic anxiety	7563	7.85%		221	5.74%		
	Bereavement	697	0.72%		90	2.34%		
	Missing	28165	29.24%		1195	31.05%		

At the end of treatment, older adults were more likely to reliably recover (52.5% vs 40.9%, *p*<0.001), reliably improve (72% vs 66.4%, *p*<0.001), and attrition was less likely (18.1% vs 31.6%, *p*<0.001) compared to working-age adults. These reliable recovery rates are slightly lower than the current national average (NHS Digital, 2020); the national rates have improved year-on-year and the sample here contributed data over a ten year period. The likelihood of reliable deterioration was similar between the age groups (8.1% vs 8.0%, *p*=0.729). Pre-post intervention effect sizes were large for both age groups although they were slightly larger for older adults (PHQ-9 change *d_rm_*=0.9; GAD-7 change *d_rm_*=0.94) compared to working-age adults (PHQ-9 change *d_rm_*=0.86; GAD-7 change *d_rm_*=0.87).

### 3.2 Association of age group with outcomes after adjusting for confounders

Before running the main analyses, the potential linear effect of age was initially checked by investigating the associations between age as a continuous variable, and age grouped into four categories (18-24 years; 25-44 years; 45-64 years and 65+ years), with the primary outcome. No evidence was found for an association between the primary outcome (reliable recovery) and age as a continuous variable: (OR(95%CI)=1.0 (0.999; 1.000)). Further, there was evidence that the odds of reliable recovery for each age group under 65 years old were lower than for those aged 65+: 16-24 (OR(95%CI)=0.59 (0.547; 0.631)), 25-44 (OR(95%CI)=0.68 (0.638; 0.728)) and 45-64 (OR(95%CI)=0.54 (0.506; 0.58)). Given the non-linear associations, age was treated as a binary variable “age group” (18-64 vs 65+ years) in all subsequent analyses.

Logistic regression models were then constructed to explore the associations between age group and reliable recovery, reliable improvement, reliable deterioration and attrition all when unadjusted, and when adjusted for different baseline confounders (see Table 3). Older adults were more likely to reach reliable recovery and reliably improve, and attrition was less likely compared to working-age adults in the univariate models. There were no differences in deterioration rates between the two age groups in the univariate model. A similar pattern of results occurred when controlling for all available confounders albeit with reduced effect estimates, the most notable difference when adjusting for all these factors was that older adults were significantly less likely to show reliable deterioration than working-age adults, see Table 3.

**Table 3:** Likelihood of each outcome for older compared to working-age adults, unadjusted and adjusted for baseline characteristics.

	Predictors and Covariates	Reliable Recovery: Odds ratio (95% Cis)	Reliable Improvement: Odds ratio (95% Cis)	Reliable deterioration: Odds ratio (95% Cis)	Attrition: Odds ratio (95% Cis)
Model 1	Older age	1.6 (1.5-1.7)	1.3 (1.21-1.4)	0.98 (0.87-1.1)	0.48 (0.44-0.52)
Model 2	+ Service, sessions	1.62 (1.51-1.73)	1.34 (1.25-1.44)	0.96 (0.85-1.08)	0.4 (0.36-0.44)
Model 3	+ PHQ9, GAD7	1.38 (1.29-1.47)	1.39 (1.29-1.49)	0.79 (0.7-0.89)	0.44 (0.4-0.49)
Model 4	+ WSAS, Phobia items	1.3 (1.22-1.4)	1.31 (1.22-1.42)	0.82 (0.73-0.93)	0.47 (0.42-0.52)
Model 5	+ demographic factors*	1.33 (1.24-1.43)	1.34 (1.24-1.45)	0.81 (0.71-0.91)	0.48 (0.43-0.53)

Propensity score matched^		1.25 (1.12-1.39)	1.31 (1.17-1.47)	0.85 (0.71-1.02)	0.53 (0.46-0.61)

* Includes: gender, problem descriptor, LTC status, ethnicity & IMD decile.

^ Uses subsample of n=3205 older adults and their matched controls. Odds ratio adjusted for all covariates.

Multilevel logistic regression models were constructed to explore service-level clustering, with results nearly identical to those of the single-level models (see Appendix D). Sensitivity analyses were conducted with observed data only, that is only cases with complete data on all confounders included in each model, in order to test the effect of using multiply imputed values for missing data in the primary analyses. Again, findings were nearly identical for the sample of cases with complete data only to those presented in Table 3 (see Appendix E).

### 3.3 Propensity score matching

In order to test the robustness of the above findings a further set of sensitivity analyses were conducted. Propensity score matching was performed on the sample of identified matched controls for the older adults on all available confounders. Of the 3226 older adults with complete data available (84%), only 21 could not be found adequate matches, resulting in a sample of 3205 adults and their matched controls. More details on the matching method and the balance on matched characteristics is presented in Appendix C. The associations between age group and outcomes in the propensity score matched groups are presented in the last row of Table 3. Results supported those of the logistic regressions, as older adults had a higher likelihood of reliable recovery, reliable improvement, and a lower likelihood treatment attrition, compared to matched controls, although the likelihood of deterioration as not lower for older adults compared to the match controls.

### 3.4 Outcomes by Problem descriptor – Depression compared to Anxiety Disorders

Subgroup analyses were performed with patients that had either a depressive disorder, or an anxiety disorder, as their diagnosis (in IAPT this is known as a ‘problem descriptor’). For this analysis, the problem descriptors PTSD, OCD, GAD, Panic and phobic anxiety disorders were collapsed together as has been done in previous studies assessing the outcomes of psychological therapy for older adults (Gould et al., 2012). Similar results to those of the primary analyses were found in subgroups with either depression (Appendix F) or an anxiety disorder (Appendix G) using imputed and observed data when adjusting for confounders: older adults were more likely to experience positive treatment outcomes (reliable recovery and reliable improvement) and both attrition and deterioration were less likely. However, after propensity score matching, older adults with depression were no longer more likely to report reliable recovery than working-age adults with depression, although they were more likely to experience reliable improvement. For patients with anxiety disorders, older adults were more likely to experience reliable recovery or reliable improvement and attrition was less likely across all models, whereas reliable deterioration did not significantly differ in any model.

### 3.5 Impact of co-occurring Long-term health conditions

**Fig. 1:**
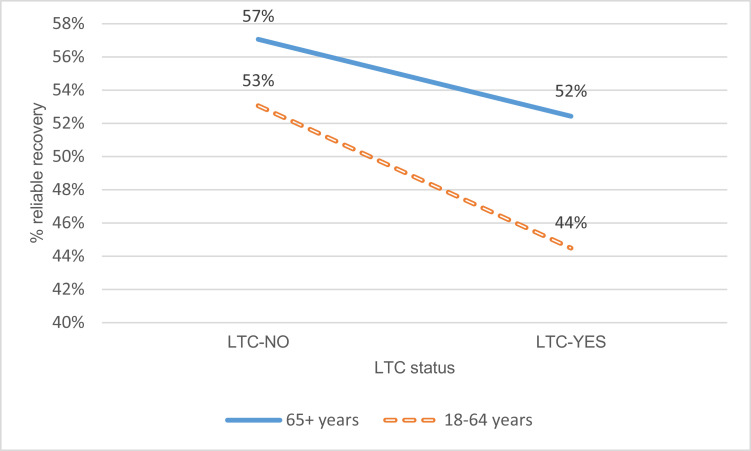
Impact of LTC status on recovery between older and working-age patients.

Analyses were then performed to explore differences in outcomes between patients with and without a self-reported LTC for both the older adult and working-age matched samples. The differential impact of LTC status by age group is presented in figure 1, and shows that LTC status had a bigger impact (reduction in the probability of reliable recovery) in working-age adults than older adults. After controlling for the same confounders as in Model 5 above, there was evidence of an association between having an LTC and reliable recovery for working-age adults (OR(95%CI)=0.79(0.66;0.94)) but not for older adults (OR(95%CI)=0.87(0.73;1.04)). Further analysis indicated that whilst there was evidence for a difference in the odds of reliable recovery associated with LTC status between groups stratified by age, there was insufficient evidence for an interaction between LTC status and age group when controlling for the main effects (OR(95%CI)=1.09(0.86;1.39)).

### 3.6 Differences in IAPT treatments

When controlling for baseline clinical and sociodemographic variables, older adults were more likely to receive only HI interventions as opposed to only LI interventions (Relative risk ratio(RRR)(95%CI)=1.12(1.03;1.22) and less likely to be stepped up (RRR(95%CI)=0.88(0.81;0.96). For patients whose last treatment was an LI intervention, compared to receiving guided self-help interventions, older adults were less likely to receive computerised interventions than working-age adults (RRR(95%CI)=0.15(0.09;0.25), but no differences between other interventions were observed. For those whose last treatment was an HI intervention, compared to receiving CBT, older adults were more likely to receive counselling (RRR(95%CI)=2.58(2.31;2.88), Interpersonal psychotherapy (IPT) (RRR(95%CI)=2.20(1.32;3.68) or another HI intervention (RRR(95%CI)=1.27(1.02;1.58) compared to working-age adults, which may indicate more choice provided to older adults. Although older adults were more likely to receive HI compared to LI treatments, on average, they received significantly fewer sessions in total (coefficient(95%CI)= -0.25(-0.42;-0.07). Further details on these analyses are presented in Appendix H.

## 4 Discussion

This study explored the difference in treatment outcomes between older adults and working-age patients in a large sample of community-based, primary care psychological treatment service attendees. We found that older people accounted for just 3.8% of patients treated at the services. This suggests that older adults were under-represented in the services that provided data here as over 65 year olds account for approximately 18% of the population (Office for National Statistics, 2019). Although older adults presented with less severe mental distress pre-treatment, once controlling for this and all other available baseline confounders, the odds of reliable recovery following treatment were 1.33 times higher for older adults compared to working-age patients. The odds of reliable improvement were higher and odds of both attrition and reliable deterioration were lower compared to working-age adults. These findings were replicated using propensity score matching. In addition, among patients with long-term health conditions alongside their common mental disorders, the odds of reliable recovery were higher for older adults than working-age adults. We also found that the effects were more pronounced in patients treated for anxiety disorders compared to those treated for depression.

The findings of this study differ from those of recent meta-analytic reviews which have suggested interventions for depression are equally effective when comparing older to working-age adults (Buckman et al., 2021b; Cuijpers et al., 2018; Haigh et al., 2018), and even that older adults suffering from anxiety disorders have worse outcomes than working-age patients (Gould et al., 2012). The contrasting findings may be due to differences in how effects were calculated. The reviews compared treatment effect sizes in studies of students (and working-age adults) to those conducted with older adult populations, or used the mean age across the included studies in meta-regressions. This is problematic as it is both inefficient and introduces potential ecological biases (i.e. making inferences about individual patients from aggregate data which may not be correct) (Fisher et al., 2017), whereby it was not possible to adjust for prognostic factors such as baseline symptom severity, which is a key indicator of treatment outcomes (Buckman et al., 2021a). This may explain some of the difference with our findings, as there were large differences in baseline symptom severity between working-age and older adults in the present study. Here we used individual patient data from a large sample, avoiding ecological biases and improving the accuracy of effect estimates. There are other reasons that the findings here may differ to those of recent reviews, for example, the reviews included clinical trials with a variety of treatment settings, whereas here the sample comes from a wholly primary care and community based mental health setting, from eight services that all follow similar protocols. In addition, the treatments may have differed; unlike the protocol-guided approaches in the RCTs (particularly as few were pragmatic RCTs; Rothwell, 2005) treatments in IAPT are perhaps more flexible and formulation-based, an approach which may be more desirable to older adults (Birdsey, 2020). This may be supported by the finding that older adults were more likely to be receiving high-intensity interventions, relative to working-age adults, after adjusting for differences in clinical presentations. Most patients will have a more detailed assessment prior to starting high-intensity therapy which may enable the clinician to more closely tailor the intervention to the individual's circumstances. This may reflect why older adults were more likely to be receiving other high-intensity interventions, such as IPT or counselling compared to working-age patients. That they received fewer sessions in total, but demonstrated better outcomes might highlight the value in more personalised and choice-informed treatment delivery. Whilst it has been suggested that co-occurring LTCs impact psychological outcomes in older people (Callahan, 2001; Laidlaw et al., 2003), this study found minimal differences in outcomes between older people with and without a reported LTC, and that the impact was more pronounced in working-age people.

## 5 Limitations

This study supports the notion that older adults who enter psychological treatment services differ from working-age patients in important ways. However, there are a number of important limitations to note. The findings presented above could be due to a selection bias whereby harder to treat older adults with more severe symptoms are less likely to enter psychological treatment services. The reasons for any such systematic selection bias cannot be ascertained from the current study. There was evidence that the outcomes for older adults were better than for working-age adults independently of all the variables available that could confound the relationships between age and outcome. However, we cannot rule out further selection biases or residual confounding; older adults with poorer prognoses might be more easily identified and referred to other services relative to working-age people with poor prognoses. Therefore, those seen for psychological treatment might systematically differ from those not seen for treatment on unmeasured factors associated with outcomes (e.g., chronicity of illness, relationship status, comorbid personality disorders and the number of previous treatments (DeRubeis et al., 2014)). An alternative explanation is that older adults might be more treatment adherent than working-age patients (Ogrodniczuk et al., 2006) and this in turn is associated with better outcomes. The current study found that the odds of attrition were lower among older patients than working-age patients, which may support this hypothesis, but the lack of adherence to treatment data means this could not be tested.

The current findings suggest that contrary to previous evidence (Gould et al., 2012), older adults with anxiety disorders may be more likely to have positive treatment outcomes than working-age patients. Here as in previous studies (Gould et al., 2012), the anxiety disorders were collapsed together, losing some potentially important nuance. This was done to ensure sufficient power for the analyses as there were relatively few older adults with some specific anxiety disorders (e.g. obsessive-compulsive disorder). Although the current sample may differ from those of clinical trials of treatments for specific anxiety disorders, it may nonetheless be more representative of the patients receiving routine treatment (Zimmerman et al., 2002).

It is noteworthy that there was a differential impact of long-term physical health conditions on treatment outcomes for the two age groups. Older adults with LTCs had marginally worse outcomes than older adults without such conditions, their outcomes were approximately equivalent to the outcomes for working-age adults without LTCs; and working-age adults with LTCs had considerably worse outcomes. One possible interpretation is that older adults are better adjusted to their LTCs than working-age adults are, and it potentially being less unexpected to have an LTC in older age. Another is that the type of LTCs suffered by older adults differ from those of working-age adults. However, there was insufficient evidence for an interaction between LTC status and age group when controlling for the main effects in the matched subgroup (this may be due to a lack of power in the analyses to appropriately test for an interaction after propensity score matching, or the relatively modest main effect of LTCs on the therapy outcome of older adults). Furthermore, we were unable to examine adjustment to LTCs or type of LTC in the present study so the role of different LTCs and age warrants further examination.

### 5.1 Clinical and research implications

Some clinicians believe psychological treatments for CMDs are less effective in older adults (Mental Health Taskforce, 2016), and older people have been found to not believe themselves to be able to benefit from psychological therapies (Laidlaw et al., 2003). The findings of the current study go some way to help dispel these views and demonstrate that psychological treatments are an appropriate option for this patient group.

To increase access, psychological treatment services may need to offer more flexible ways of working to support older people with medical or mobility issues that make it more difficult for them to attend services in-person. Qualitative research should explore the potential reasons why clinicians may choose not to refer older adults to psychological therapies, and why some older adults decline psychological therapies, in order to understand the barriers to access for this group. Improving the visibility of non-stigmatising information about psychological treatment services in places more likely to be seen by or attended by older adults might also increase self-referral rates.

In addition, future studies should investigate factors which might elucidate mechanisms of the effects found here. For example, it has been suggested that there are differences in CMD symptom constellations between older and working-age patients (Bodner et al., 2018) which may be more amenable to change with psychological therapy, or potentially older adults interpret symptom scales differently resulting in larger reported change in symptoms. Further, whether age moderates the adjustments patients make to cope with their LTCs should be explored, as should therapeutic interventions or adjuncts to psychological therapies which may better support patients with LTCs. These should include interventions that may be effective at supporting patients with residual symptoms at the end of their treatment, for example well-being therapy (Fava, 2016), as this has been shown to reduce the risk of relapse and poorer long-term prognoses (Buckman et al., 2018).

## 6 Conclusion

The findings from this study support the case for increased access to psychological treatment services for older adults and counters the narrative that older people are less likely to benefit from psychological therapy, or views that they may be less psychologically flexible or unable to change. Our findings suggest these differences cannot be explained by the fact that on average older adults had less severe presentations and attended more treatment sessions. Greater understanding of the reasons why older people might have better therapy outcomes than working-age adults, particularly for those with anxiety disorders, could inform means of improving outcomes in other age and diagnostic groups. That the presence of LTCs did not appear to have the same impact on outcomes in older people as they did for working-age patients suggests that having a LTC should not be seen as a barrier to referring older adults and that greater attention might be paid to working-age adults with LTCs.

## Author contributions

RS contributed to the study design, literature search, conducted the analysis, and drafted the initial version of the manuscript and appendix. JB contributed to the study design, literature search, analysis plan, data interpretation, drafted the initial version of the manuscript and appendix, and final approval. JS contributed to the study design, literature search, analysis plan, data interpretation, supported the drafting of the manuscript and final approval. JL contributed to the study design, data acquisition, data interpretation and final approval. EA contributed to the study design, data acquisition, data interpretation, drafting of the manuscript and final approval. AJ contributed to the data interpretation, drafting of the manuscript and appendix, and final approval. GL contributed to the data interpretation and final approval. JC contributed to the study design, analysis plan, data interpretation, drafting of the manuscript, and final approval. SP contributed to the study design, analysis plan, data interpretation, drafting of the manuscript, and final approval.

## Financial support

This work was supported by the Wellcome Trust (Grant Code 201292/Z/16/Z), the National Institute for Health Research University College London Hospitals Biomedical Research Centre, University College London, and the Alzheimer's Society (grant code: 457 (AS-PG-18-013)). None of these funders had any role in the study design, collection, analysis or interpretation of the data, writing the manuscript, or the decision to submit the paper for publication.

## Declaration of Competing Interest

None to declare.

## Appendix Supplementary materials

Image, application 1

## References

[bib0001] Amati F., Banks C., Greenfield G., Green J. (2018). Predictors of outcomes for patients with common mental health disorders receiving psychological therapies in community settings: a systematic review. J. Public Health (Bangkok)..

[bib0002] Austin P.C. (2011). An introduction to propensity score methods for reducing the effects of confounding in observational studies. Multivariate Behav. Res..

[bib0003] Birdsey N. (2020). Integrating CBT and CFT within a case formulation approach to reduce depression and anxiety in an older adult with a complex mental and physical health history: a single case study. Cogn. Behav. Ther..

[bib0004] Bodner E., Palgi Y., Wyman M.F. (2018). Contemporary Perspectives on Ageism.

[bib0005] Buckman J.E.J., Saunders R., Cohen Z.D., Barnett P., Clarke K., Ambler G., DeRubeis R.J., Gilbody S., Hollon S.D., Kendrick T., Watkins E., Wiles N., Kessler D., Richards D., Sharp D., Brabyn S., Littlewood E., Salisbury C., White I.R., Lewis G., Pilling S. (2021). The contribution of depressive ‘disorder characteristics’ to determinations of prognosis for adults with depression: an individual patient data meta-analysis. Psychol. Med..

[bib0006] Buckman J.E.J., Saunders R., Stott J., Arundell L.-L., O'Driscoll C., Davies M., Eley T.C., Hollon S.D., Kendrick T., Ambler G., Cohen Z.D., Watkins E.R., Gilbody S., Wiles N.J., Kessler D., Richards D., Brabyn S., Littlewood E., DeRubeis R.J., Lewis G., Pilling S. (2021). The role of Age, Gender, and Marital Status in prognosis for adults with depression: An Individual Patient Data Meta- analysis. Epidemiol. Psychiatr. Sci..

[bib0007] Buckman J.E.J., Underwood A., Clarke K., Saunders R., Hollon S.D., Fearon P., Pilling S. (2018). Risk factors for relapse and recurrence of depression in adults and how they operate: A four-phase systematic review and meta-synthesis. Clin. Psychol. Rev..

[bib0008] Byers A.L., Yaffe K. (2011). Depression and risk of developing dementia. Nat. Rev. Neurol..

[bib0009] Callahan C.M. (2001). Quality improvement research on late life depression in primary care. Med. Care.

[bib0010] Carvalho A.F., Sharma M.S., Brunoni A.R., Vieta E., Fava G.A. (2016). The safety, tolerability and risks associated with the use of newer generation antidepressant drugs: a critical review of the literature. Psychother. Psychosom..

[bib0011] Catarino A., Bateup S., Tablan V., Innes K., Freer S., Richards A., Stott R., Hollon S.D., Chamberlain S.R., Hayes A., Blackwell A.D. (2018). Demographic and clinical predictors of response to internet-enabled cognitive–behavioural therapy for depression and anxiety. BJPsych Open.

[bib0012] Chand S.P., Grossberg C.T. (2013). How to adapt cognitive-behavioral therapy for older adults. Curr. Psychiatr..

[bib0013] Clark D.M. (2018). Realizing the mass public benefit of evidence-based psychological therapies: the IAPT program. Annu. Rev. Clin. Psychol..

[bib0014] Clark D.M., Canvin L., Green J., Layard R., Pilling S., Janecka M. (2018). Transparency about the outcomes of mental health services (IAPT approach): an analysis of public data. Lancet.

[bib0016] Community and Mental Health team, N.D. (2016).

[bib0017] Cosci F., Fava G.A., Sonino N. (2015). Mood and anxiety disorders as early manifestations of medical illness: a systematic review. Psychother. Psychosom..

[bib0018] Cuijpers P., Karyotaki E., Reijnders M., Huibers M.J.H. (2018). Who benefits from psychotherapies for adult depression? A meta-analytic update of the evidence. Cogn. Behav. Ther..

[bib0019] Cuijpers P., Noma H., Karyotaki E., Vinkers C.H., Cipriani A., Furukawa T.A. (2020). A network meta-analysis of the effects of psychotherapies, pharmacotherapies and their combination in the treatment of adult depression. World Psychiatry.

[bib0020] DeRubeis R.J., Cohen Z.D., Forand N.R., Fournier J.C., Gelfand L.A., Lorenzo-Luaces L. (2014). The personalized advantage index: translating research on prediction into individualized treatment recommendations. A demonstration. PLoS One.

[bib0021] Djernes J.K. (2006). Prevalence and predictors of depression in populations of elderly: a review. Acta Psychiatr. Scand..

[bib0022] Evans M., Mottram P. (2000). Diagnosis of depression in elderly patients. Adv. Psychiatr. Treat..

[bib0023] Fava G.A. (2016). Well-being therapy: current indications and emerging perspectives. Psychother. Psychosom..

[bib0024] Fava G.A., Cosci F., Sonino N. (2016). Current psychosomatic practice. Psychother. Psychosom..

[bib0025] Fisher D.J., Carpenter J.R., Morris T.P., Freeman S.C., Tierney J.F. (2017). Meta-analytical methods to identify who benefits most from treatments : daft, deluded, or deft approach ? most from particular treatments or other broad approaches used for testing such. BMJ Br. Med. J..

[bib0026] Gould R.L., Coulson M.C., Howard R.J. (2012). Efficacy of cognitive behavioral therapy for anxiety disorders in older people: a meta-analysis and meta-regression of randomized controlled trials. J. Am. Geriatr. Soc..

[bib0027] Gowling S., Persson J., Holt G., Ashbourne S., Bloomfield J., Shortland H., Bate C. (2016). Richmond wellbeing service access strategy for older adults. BMJ Qual. Improv. Reports.

[bib0028] Haigh E.A.P., Bogucki O.E., Sigmon S.T., Blazer D.G. (2018). Depression among older adults: a 20-year update on five common myths and misconceptions. Am. J. Geriatr. Psychiatry.

[bib0029] He W., Goodkind D., Kowal P. (2016).

[bib0030] Kazmi H., Walker Z., Booij J., Khan F., Shah S., Sudre C.H., Buckman J.E.J., Schrag A.-E. (2021). Late onset depression: dopaminergic deficit and clinical features of prodromal Parkinson's disease: a cross-sectional study. J. Neurol. Neurosurg. Psychiatry.

[bib0031] Laidlaw K., Thompson L.W., Gallagher-Thompson D., Dick-Siskin L. (2003).

[bib0032] Maust D.T., Blow F.C., Wiechers I.R., Kales H.C., Marcus S.C. (2017). National trends in antidepressant, benzodiazepine, and other sedative-hypnotic treatment of older adults in psychiatric and primary care. J. Clin. Psychiatry.

[bib0033] McHugh R.K., Whitton S.W., Peckham A.D., Welge J.A., Otto M.W. (2013). Patient preference for psychological vs pharmacologic treatment of psychiatric disorders: a meta-analytic review. J. Clin. Psychiatry.

[bib0034] Mental Health Taskforce, 2016. The Five Year Forward View for Mental Health.

[bib0035] NHS Digital, 2020. Psychological Therapies, Annual report on the use of IAPT services 2019-20.

[bib0036] NHS Digital, 2019. Psychological Therapies, Annual report on the use of IAPT services 2018-19.

[bib0037] NICE (2011). Common mental health problems: identification and pathways to care. Clin. Guidel..

[bib0038] Office for National Statistics, 2019. Overview of the UK Population, Overview of the UK population August 2019.

[bib0039] Ogrodniczuk J.S., Piper W.E., Joyce A.S. (2006). Treatment compliance in different types of group psychotherapy: exploring the effect of age. J. Nerv. Ment. Dis..

[bib0040] Rothwell P.M. (2005). Subgroup analysis in randomised controlled trials : importance, indications, and interpretation. Lancet.

[bib0041] Royston P., White I.R. (2011). Multiple Imputation by Chained Equations (MICE): Implementation in Stata. J. Stat. Softw..

[bib0042] Sanglier T., Saragoussi D., Milea D., Auray J.P., Valuck R.J., Tournier M. (2011). Comparing antidepressant treatment patterns in older and younger adults: A claims database analysis. J. Am. Geriatr. Soc..

[bib0043] Saunders R., Buckman J.E.J., Cape J., Fearon P., Leibowitz J., Pilling S. (2019). Trajectories of depression and anxiety symptom change during psychological therapy. J. Affect. Disord..

[bib0044] Saunders R., Cape J., Leibowitz J., Aguirre E., Jena R., Cirkovic M., Wheatley J., Main N., Pilling S., Buckman J.E.J. (2020). Improvement in IAPT outcomes over time: are they driven by changes in clinical practice?. Cogn. Behav. Ther..

[bib0045] Saz P., Dewey M.E. (2001). Depression, depressive symptoms and mortality in persons aged 65 and over living in the community: a systematic review of the literature. Int. J. Geriatr. Psychiatry.

[bib0046] Stanley M.A., Wilson N.L., Novy D.M., Rhoades H.M., Wagener P.D., Greisinger A.J., Cully J.A., Kunik M.E. (2009). Cognitive behavior therapy for generalized anxiety disorder among older adults in primary care a randomized clinical trial. JAMA - J. Am. Med. Assoc..

[bib0047] StataCorp (2019). Stata Statistical Software: Release 16. Stata Stat. Softw..

[bib0048] Tamblyn R., Bates D.W., Buckeridge D.L., Dixon W., Forster A.J., Girard N., Haas J., Habib B., Kurteva S., Li J., Sheppard T. (2019). Multinational comparison of new antidepressant use in older adults: a cohort study. BMJ Open.

[bib0049] Unützer J., Patrick D.L., Diehr P., Simon G., Grembowski D., Katon W. (2000). Quality adjusted life years in older adults with depressive symptoms and chronic medical disorders. Int. Psychogeriatrics.

[bib0050] World Health Organization, 2016. Mental health and older adults [WWW Document]. Media Cent.

[bib0051] Zimmerman M., Mattia J.I., Posternak M.A. (2002). Are subjects in pharmacological treatment trials of depression representative of patients in routine clinical practice?. Am. J. Psychiatry.

