# Supplementary material for: Older adults respond better to psychological therapy than working-age adults: evidence from a large sample of mental health service attendees
Source: J Affect Disord. 2021 Nov 1;294:85–93. doi: 10.1016/j.jad.2021.06.084 (PMC8411661; doi:10.1016/j.jad.2021.06.084)
Supplement: Supplementary file 1 [file mmc1.pdf]

Supplementary materials to:

Older adults respond better to psychological therapy than working-age adults: evidence from a large cohort of mental health service attendees.

Content:

Appendix A: Patient flow diagram (Page 2)

Appendix B: Anxiety Disorder Specific Measures used by IAPT. (Page 3)

Appendix C: Propensity score matching – methods. (Page 4)

Appendix D: Multilevel logistic regression models – imputed data. (Page 6)

Appendix E: Odds ratios for Older Adults on outcomes – observed data. (Page 7)

Appendix F: Analyses for patients with depression. (Page 9)

Appendix G: Analyses for patients with anxiety disorders. (Page 11)

Appendix H: Analyses of IAPT treatment received. (Page 13)

References. (Page 15)

Appendix A: Patient flow diagram

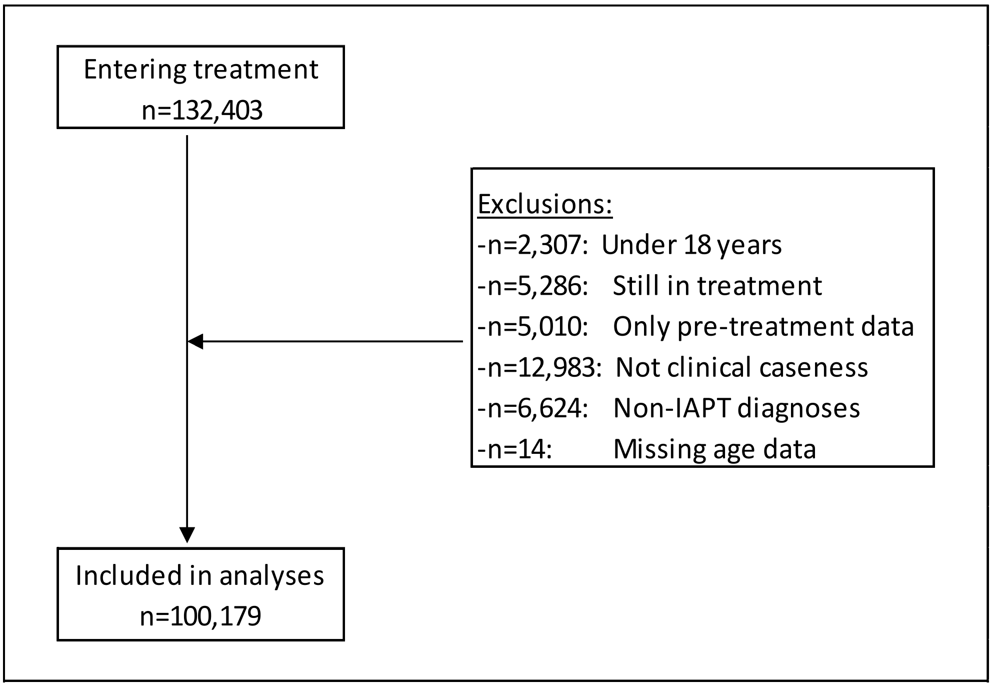

Figure A1: Patient flow diagram.

Appendix B: Anxiety Disorder Specific Measures used by IAPT services.

Table B1. Recommended ADSMs (adapted from NHS Digital, 2016).

Problem descriptor	Recommended ADSM	Threshold for caseness	Threshold for reliable change
Agoraphobia	Mobility Inventory (Chambless et al., 1985)	2.3 (for version 1.5)	0.73 (for version 1.5)
Health anxiety	Health Anxiety Inventory (Salkovskis et al., 2002)	18	4
Obsessive compulsive disorder (OCD)	Obsessive Compulsive Inventory (Foa et al., 1998)	40	32
Panic disorder	Panic Disorder Severity Scale (Shear et al., 2001)	-	-
Post-traumatic stress disorder (PTSD)	Impact of Events Scale (IES-R) (Creamer et al., 2003)	33	9
Social anxiety disorder	Social Phobia Inventory(Connor et al., 2000)	19	10

Supplementary Table B1 presents the recommended Anxiety Disorder Specific Measures (ADSMs) used in IAPT services for each anxiety disorder problem descriptor. The table also includes the threshold which indicates clinical caseness on each measure as well as the number of points of change on the measure which are used to indicate reliable change (improvement/deterioration). Note that the PDSS does not have a threshold for caseness or reliable change and instead the GAD-7 is used in the calculation of key IAPT outcomes for these individuals.

Appendix C: Propensity score matching analysis.

Matching was performed on all available clinical and demographic variables, using “psmatch2” (Leuven and Sianesi, 2003) in Stata16 (StataCorp, 2017). Only cases with complete data on continuous covariates were included, but missing data for gender, diagnosis, deprivation, ethnicity and LTC status was dummy coded so that cases with missing data on these covariates could be used in the matching. The caliper was set at 0.001 and the first nearest neighbour was identified for each older adult in the working-age adult pool.

Adequate matches could not be found for 21 older adults. After excluding these cases, 3205 older adults and their matched controls were retained for the final set of analyses. The quality of the matching was explored by comparing the older adults with their matched controls on their characteristics to assess balance between the matched groups. The results of these are presented in Table C1 below and indicate a good balance of matching, with no significant differences in clinical and demographic characteristics between older adults and their matched controls. Controls that were identified as matches for more than one older adult (that is they were identified as being very similar to two different older adult cases) were weighted in the analysis by the number of times they were found to be a match (maximum weighting in current analysis was three).

Table C1. Balance between Older Adults and matched controls.

Patient characteristic	18-64		65+		t	p
	mean	sd	mean	sd		
PHQ-9	14.07	5.61	14.01	5.52	0.37	0.708
GAD-7	12.96	4.51	12.89	4.62	0.66	0.509
WSAS-item 2	3.37	2.41	3.29	2.49	1.18	0.239
WSAS-item 3	3.81	2.47	3.70	2.61	1.69	0.091
WSAS-item 4	3.40	2.53	3.27	2.53	1.94	0.053
WSAS-item 5	3.15	2.40	3.03	2.53	1.84	0.066
Agoraphobia item	2.41	2.64	2.35	2.66	0.87	0.387
Social phobia item	2.57	2.44	2.47	2.55	1.61	0.108
Specific phobia item	2.28	2.75	2.21	2.81	0.98	0.328
Number LI sessions	3.15	2.95	3.12	3.06	0.44	0.658
Number HI sessions	4.39	5.18	4.26	5.06	1.02	0.306
Waiting time (days) - referral to assessment	24.80	31.54	25.24	30.55	-0.55	0.580
Waiting time (days) - assessment to treatment	69.77	75.28	69.12	75.73	0.34	0.735

Patient characteristic		18-64 n	18-64 %	65+ n	65+ %	chi	p
Gender	Male	946	31.56%	991	30.92%	0.50	0.78
	Female	2041	68.10%	2201	68.67%		
	Missing	10	0.33%	13	0.41%		
LTC Case	No	936	31.23%	991	30.92%	0.08	0.962
	Yes	1443	48.15%	1553	48.46%		
	Missing	618	20.62%	661	20.62%		
Ethnicity (ONS)	White	2308	77.01%	2494	77.82%	4.28	0.639
	Mixed	52	1.74%	45	1.40%		
	Asian	208	6.94%	202	6.30%		
	Black	184	6.14%	183	5.71%		
	Chinese	7	0.23%	8	0.25%		
	Other	84	2.80%	85	2.65%		
	Missing	154	5.14%	188	5.87%		
Psychotropic medication	Prescribed - not taking	110	3.67%	144	4.49%	4.51	0.211
	Prescribed and taking	1211	40.41%	1246	38.88%		
	Not prescribed	1433	47.81%	1531	47.77%		
	Missing	243	8.11%	284	8.86%		
Problem descriptor	Depression	1305	43.54%	1401	43.71%	5.59	0.588
	MADD	250	8.34%	236	7.36%		
	GAD	491	16.38%	545	17.00%		
	OCD	32	1.07%	29	0.90%		
	PTSD	57	1.90%	50	1.56%		
	Phobic anxiety	197	6.57%	211	6.58%		
	Bereavement	84	2.80%	77	2.40%		
	Missing	581	19.39%	656	20.47%		
Index of Multiple Deprivation (IMD) Decile	1	250	8.34%	254	7.93%	3.60	0.963
	2	680	22.69%	715	22.31%		
	3	566	18.89%	588	18.35%		
	4	319	10.64%	328	10.23%		
	5	279	9.31%	311	9.70%		
	6	220	7.34%	246	7.68%		
	7	181	6.04%	211	6.58%		
	8	213	7.11%	234	7.30%		
	9	116	3.87%	128	3.99%		
	10	49	1.63%	44	1.37%		
	Missing	124	4.14%	146	4.56%		

Notes: This table presents a comparison between the included older adults and their matched controls identified in the working-age adult pool. There were no significant differences on any covariate between the groups, indicating good matching and balance.

Appendix D: Multilevel logistic regression models - imputed dataset.

Table D1. Results from multilevel logistic regression models.

	Reliable Recovery		Reliable Improvement	
	OR (95% CI)	ICC	OR (95% CI)	ICC
Model 1	-	-	-	-
Model 2	1.62 (1.51-1.73)	0.018	1.34 (1.25-1.44)	0.020
Model 3	1.38 (1.29-1.47)	0.029	1.39 (1.29-1.49)	0.020
Model 4	1.3 (1.22-1.4)	0.034	1.31 (1.22-1.42)	0.015
Model 5	1.32 (1.23-1.42)	0.031	1.35 (1.25-1.45)	0.013

	Reliable Deterioration		Attrition	
	OR (95% CI)	ICC	OR (95% CI)	ICC
Model 1	-	-	-	-
Model 2	0.96 (0.85-1.08)	0.033	0.4 (0.36-0.44)	0.105
Model 3	0.79 (0.7-0.89)	0.045	0.44 (0.4-0.49)	0.099
Model 4	0.82 (0.73-0.93)	0.038	0.47 (0.42-0.52)	0.096
Model 5	0.8 (0.7-0.9)	0.040	0.48 (0.43-0.53)	0.097

Note: Model 1 (‘older adults’ only is empty) as all models include ‘service’ as a clustering variable.

This table presents the odds ratios (OR) and 95% confidence intervals (95% CI) for older adults in a series of multilevel logistic regression models where service was included as a second level of analysis, to take into account of the service-level clustering of the data. The odds ratios are almost identical to the primary analysis (single-level) logistic regression models presented in Table 2 of the manuscript. The Intraclass Correlation Coefficient (ICC) values show that less than 4.5% of the likelihood of reliable recovery, improvement or deterioration was explained by between-service differences. Between-service differences explained up to 10% of the likelihood of attrition, suggesting more service level variation, although the odds ratios were almost identical to the single-level models.

Appendix E: Odds ratios for Older Adults on outcomes - observed data.

Table E1. Results from single-level logistic regression models (observed data).

		Reliable Recovery: Odds ratio (95% Cis)	Reliable Improvement: Odds ratio (95% Cis)	Reliable Deterioration: Odds ratio (95% Cis)	Attrition: Odds ratio (95% Cis)
Model 1	Older age	1.6 (1.50-1.70)	1.30 (1.21-1.40)	0.98 (0.87-1.10)	0.48 (0.44-0.52)
Model 2	+ Service, sessions	1.61 (1.51-1.72)	1.34 (1.24-1.44)	0.96 (0.85-1.09)	0.40 (0.36-0.44)
Model 3	+ PHQ9, GAD7	1.38 (1.29-1.47)	1.38 (1.28-1.49)	0.79 (0.70-0.89)	0.45 (0.40-0.49)
Model 4	+ WSAS, Phobia items	1.29 (1.20-1.39)	1.32 (1.22-1.44)	0.82 (0.72-0.95)	0.50 (0.45-0.56)
Model 5	+ demographic factors*	1.32 (1.22-1.42)	1.37 (1.26-1.49)	0.80 (0.70-0.92)	0.52 (0.47-0.58)

* Includes: gender, problem descriptor, LTC status, ethnicity & IMD decile.

Table E1 presents results from the logistic regression models using cases with observed/complete data in each model. Results are very similar to those presented for the imputed data in the primary analyses (Table 2 in main manuscript). The small differences occur in the final models (model 5) where categorical demographics with missing data were dummy coded and the response of ‘missing’ was used as a value in model. However, results were still very similar across the dummy-coded and imputed models.

Table E2 presents results from multilevel logistic models clustering by service. Results appear near identical to the single-level models in Table E1. Between-service differences explained limited variation in outcome.

Table E2. Results from multilevel logistic regression models (observed data).

	Reliable Recovery		Reliable Improvement	
	**OR (95% CI)**	**ICC**	**OR (95% CI)**	**ICC**
Model 1	-	-	-	-
Model 2	1.61 (1.51-1.72)	0.001	1.34 (1.24-1.44)	0.001
Model 3	1.38 (1.29-1.47)	0.003	1.38 (1.28-1.49)	0.001
Model 4	1.29 (1.2-1.39)	0.003	1.32 (1.22-1.43)	0.001
Model 5	1.32 (1.22-1.42)	0.002	1.37 (1.26-1.49)	0.001
	**Reliable Deterioration**		**Attrition**	
	**OR (95% CI)**	**ICC**	**OR (95% CI)**	**ICC**
Model 1	-	-	-	-
Model 2	0.96 (0.85-1.09)	0.004	0.4 (0.36-0.44)	0.043
Model 3	0.79 (0.7-0.9)	0.007	0.45 (0.4-0.49)	0.038
Model 4	0.83 (0.72-0.95)	0.008	0.5 (0.45-0.56)	0.031
Model 5	0.8 (0.7-0.92)	0.009	0.52 (0.47-0.58)	0.024

Appendix F: Analyses for patients with depression.

Table F1. Results from logistic regression models (imputed data – depression cases).

		Reliable Recovery: Odds ratio (95% Cis)	Reliable Improvement: Odds ratio (95% Cis)	Reliable Deterioration: Odds ratio (95% Cis)	Attrition: Odds ratio (95% Cis)
Model 1	Older age	1.53 (1.38-1.7)	1.29 (1.15-1.45)	0.9 (0.74-1.09)	0.53 (0.47-0.61)
Model 2	+ Service, sessions	1.54 (1.38-1.71)	1.33 (1.18-1.49)	0.89 (0.74-1.09)	0.46 (0.39-0.53)
Model 3	+ PHQ9, GAD7	1.26 (1.13-1.41)	1.36 (1.21-1.53)	0.71 (0.58-0.87)	0.51 (0.44-0.59)
Model 4	+ WSAS, Phobia items	1.2 (1.07-1.34)	1.29 (1.14-1.45)	0.75 (0.61-0.91)	0.54 (0.46-0.63)
Model 5	+ demographic factors*	1.24 (1.11-1.39)	1.32 (1.17-1.49)	0.75 (0.62-0.92)	0.56 (0.48-0.65)

* Includes: gender, problem descriptor, LTC status, ethnicity & IMD.

Tables F1 and F2 present the odds ratios for older adults in logistic regression models of the four outcomes of interest using only cases with depression, using imputed data and observed data only, respectively. Results are very similar and indicate older adults with depression have a higher likelihood of reliable recovery and improvement, and lower likelihood of deterioration and attrition compared with working-age adults with depression. The differences in odds ratios between these tables and Table 3 (main manuscript) suggest there might be a smaller effect of age group on therapy outcomes for patients with depression.

Table F2. Results from logistic regression models (observed data - depression cases).

		Reliable Recovery: Odds ratio (95% Cis)	Reliable Improvement: Odds ratio (95% Cis)	Reliable Deterioration: Odds ratio (95% Cis)	Attrition: Odds ratio (95% Cis)
Model 1	Older age	1.53 (1.38-1.7)	1.29 (1.15-1.45)	0.9 (0.74-1.09)	0.53 (0.47-0.61)
Model 2	+ Service, sessions	1.54 (1.38-1.71)	1.33 (1.18-1.49)	0.9 (0.74-1.09)	0.46 (0.39-0.53)
Model 3	+ PHQ9, GAD7	1.26 (1.13-1.41)	1.36 (1.21-1.53)	0.71 (0.59-0.87)	0.51 (0.44-0.59)
Model 4	+ WSAS, Phobia items	1.16 (1.03-1.3)	1.27 (1.12-1.43)	0.77 (0.63-0.94)	0.55 (0.47-0.64)
Model 5	+ demographic factors*	1.2 (1.07-1.35)	1.33 (1.18-1.51)	0.76 (0.62-0.93)	0.57 (0.49-0.67)

** Includes: gender, problem descriptor, LTC status, ethnicity & IMD decile.*

Propensity score matching was then performed using the same method as presented in the primary analyses. Of n=1456 older adults with depression recorded as their problem descriptor, n=1407 (96.6%) had complete data on all covariates, and of these n=29 (2.1%) could not be found adequate matches and were off-support. Logistic regression models were then built on the matched sample and results presented in Table F3. Findings show age group was not associated with the likelihood of either reliable recovery or reliable deterioration for adults with depression. However, reliable improvement was more likely, and attrition less likely, in older adults with depression.

Table F3. Results from logistic regression models (matched sample - depression cases).

		Reliable Recovery: Odds ratio (95% Cis)	Reliable Improvement: Odds ratio (95% Cis)	Reliable Deterioration: Odds ratio (95% Cis)	Attrition: Odds ratio (95% Cis)
Model 1	Older age	1.08 (0.93-1.25)	1.24 (1.05-1.46)	0.9 (0.69-1.17)	0.65 (0.54-0.78)
Model 2	+ Service, sessions	1.08 (0.93-1.26)	1.25 (1.06-1.47)	0.89 (0.68-1.16)	0.61 (0.49-0.75)
Model 3	+ PHQ9, GAD7	1.1 (0.94-1.28)	1.24 (1.05-1.47)	0.89 (0.68-1.16)	0.59 (0.48-0.73)
Model 4	+ WSAS, Phobia items	1.08 (0.93-1.27)	1.24 (1.05-1.46)	0.89 (0.67-1.17)	0.6 (0.49-0.74)
Model 5	+ demographic factors*	1.09 (0.93-1.27)	1.24 (1.05-1.47)	0.89 (0.67-1.17)	0.6 (0.49-0.74)

** Includes: gender, problem descriptor, LTC status, ethnicity & IMD decile.*

Appendix G: Analyses for patients with anxiety disorders.

Table G1. Results from logistic regression models (imputed data – anxiety disorder cases).

		Reliable Recovery: Odds ratio (95% Cis)	Reliable Improvement: Odds ratio (95% Cis)	Reliable Deterioration: Odds ratio (95% Cis)	Attrition: Odds ratio (95% Cis)
Model 1	Older age	1.81 (1.58-2.08)	1.41 (1.2-1.66)	0.98 (0.74-1.29)	0.52 (0.43-0.63)
Model 2	+ Service, sessions	1.74 (1.51-2)	1.44 (1.21-1.7)	0.97 (0.73-1.28)	0.4 (0.33-0.49)
Model 3	+ PHQ9, GAD7	1.55 (1.34-1.79)	1.46 (1.23-1.73)	0.87 (0.65-1.15)	0.45 (0.37-0.56)
Model 4	+ WSAS, Phobia items	1.45 (1.26-1.68)	1.37 (1.16-1.62)	0.89 (0.67-1.19)	0.48 (0.39-0.59)
Model 5	+ demographic factors*	1.54 (1.33-1.78)	1.41 (1.19-1.68)	0.89 (0.67-1.19)	0.5 (0.41-0.62)

* Includes: gender, problem descriptor, LTC status, ethnicity & IMD decile.

Tables G1 and G2 present the odds ratios for older adults in logistic regression models of the four outcomes of interest using only cases with an anxiety disorder, using imputed data and observed data only, respectively. For these analyses, individuals with a problem descriptor of GAD, OCD, panic disorder, social anxiety disorder and PTSD were considered as having an anxiety disorder. Results were very similar to those in the primary analyses and indicate that older adults with an anxiety disorder have a higher likelihood of reliable recovery and improvement, and lower likelihood of deterioration and attrition compared with working-age adults with an anxiety disorder. The differences in odds ratios from these analyses compared to the primary analyses (irrespective of diagnosis) suggest that the effect of age group on treatment outcomes is more pronounced in patients with an anxiety disorder.

Table G2. Results from logistic regression models (observed data - anxiety disorder cases).

		Reliable Recovery: Odds ratio (95% Cis)	Reliable Improvement: Odds ratio (95% Cis)	Reliable Deterioration: Odds ratio (95% Cis)	Attrition: Odds ratio (95% Cis)
Model 1	Older age	1.81 (1.58-2.08)	1.41 (1.2-1.66)	0.98 (0.74-1.29)	0.52 (0.43-0.63)
Model 2	+ Service, sessions	1.74 (1.51-2)	1.43 (1.21-1.7)	0.97 (0.73-1.28)	0.4 (0.33-0.49)
Model 3	+ PHQ9, GAD7	1.55 (1.34-1.8)	1.46 (1.23-1.72)	0.87 (0.65-1.15)	0.45 (0.37-0.56)
Model 4	+ WSAS, Phobia items	1.47 (1.27-1.71)	1.39 (1.17-1.65)	0.9 (0.67-1.2)	0.49 (0.4-0.61)
Model 5	+ demographic factors*	1.55 (1.33-1.8)	1.46 (1.23-1.75)	0.85 (0.63-1.14)	0.49 (0.4-0.61)

** Includes: gender, problem descriptor, LTC status, ethnicity & IMD decile.*

Propensity score matching was performed using the same method as presented in the main analyses. Of n=859 older adults with an anxiety disorder recorded as their problem descriptor, n=838 (97.6%) had complete data on all covariates, and of these n=13 (1.6%) could not be found adequate matches and were off-support. Logistic regression models were then built on the matched sample and results are presented in Table G3. Findings show that older adults with an anxiety disorder had higher odds of reliable recovery and improvement, and lower odds of deterioration and attrition, relative to working-age adults with an anxiety disorder.

Table G3. Results from logistic regression models (matched sample - anxiety disorder cases).

		Reliable Recovery: Odds ratio (95% Cis)	Improvement: Odds ratio (95% Cis)	Reliable Deterioration: Odds ratio (95% Cis)	Attrition: Odds ratio (95% Cis)
Model 1	Older age	1.62 (1.33-1.97)	1.49 (1.19-1.87)	0.69 (0.48-1.01)	0.48 (0.38-0.61)
Model 2	+ Service, sessions	1.65 (1.35-2.01)	1.55 (1.23-1.95)	0.68 (0.47-1)	0.4 (0.3-0.53)
Model 3	+ PHQ9, GAD7	1.69 (1.38-2.07)	1.57 (1.25-1.98)	0.68 (0.46-1)	0.39 (0.3-0.52)
Model 4	+ WSAS, Phobia items	1.69 (1.38-2.08)	1.58 (1.25-1.99)	0.68 (0.47-1)	0.39 (0.3-0.53)
Model 5	+ demographic factors*	1.72 (1.4-2.12)	1.59 (1.26-2.02)	0.7 (0.48-1.04)	0.38 (0.28-0.51)

** Includes: gender, problem descriptor, LTC status, ethnicity & IMD decile.*

Appendix H: Analyses of IAPT treatment received.

The proportion of older adults who were stepped up (25%) was lower than the number of working-age adults stepped up (31%), and a higher proportion received only LI or HI interventions (Table H1). Adjusted multinomial regression models observed that older adults were significantly more likely to receive only HI treatments and were significantly less likely to be stepped up instead of receiving LI only when compared to working age adults.

Table H1. IAPT pathway differences between Older and working-age adults.

	**18-64**		**65+**	
	**n**	**%**	**n**	**%**
LI only	38980	40.47%	1713	44.51%
HI only	22593	23.45%	981	25.49%
Stepped Down	4066	4.22%	161	4.18%
Stepped Up	30167	31.32%	960	24.94%
Missing	524	0.54%	34	0.88%
Total	96330		3849	

	**Relative Risk Ratios for 'Older Adult' (95% CIs)***
HI only (vs LI only)	1.12 (1.03-1.22)
Stepped Down (vs LI only)	1.06 (0.89-1.25)
Stepped Up (vs LI only)	0.88 (0.81-0.96)

Notes: * Adjusted for all clinical and sociodemographic variables

Older adults on average received fewer treatment sessions in total, compared to working-age adults (coefficient(95%CI)=-0.25(-0.42;-0.07)). The histogram presented in figure H2 shows that a higher proportion of the older adult sample received between 4 and 9 sessions of IAPT treatment, whereas a higher proportion working-age adults received either 2 or 3 sessions, or more than 10 sessions.

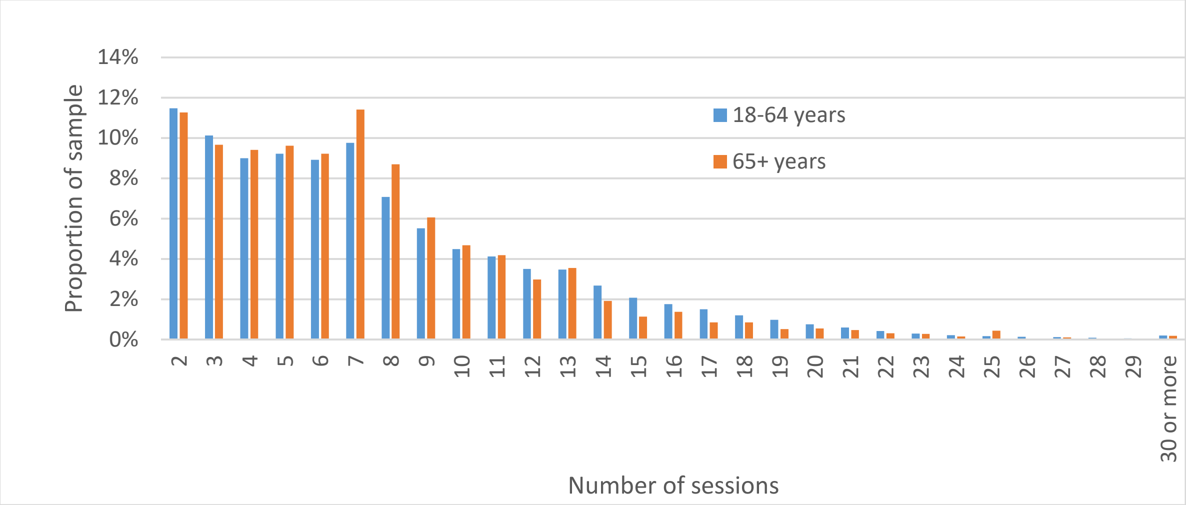

Figure 2: Distribution of sessions received between older and working-age adults

Finally, differences in the last treatment type recorded for older and working-age adults is presented in Table H3. A range of interventions were recorded as the last treatment received, and noticeably CBT was less likely for older adults. Adjusted multinomial regression models were constructed separately for patients with LI interventions as their last treatment and those with HI interventions (see Table H4). With regard to LI interventions, the only difference between older and working-age adults was for computerised interventions where older adults were much less likely to be in receipt compared to guided (facilitated) self-help interventions. However, compared to receiving CBT, older adults were more likely to be receiving counseling, interpersonal therapy (IPT) and other HI interventions than working-age adults.

Table H3. Differences between Older and working-age adults on the last treatment type received.

	**18-64**		**65+**	
	**n**	**%**	**n**	**%**
Guided Self-Help	21,756	22.58%	1,043	27.10%
Self-Help (book)	7,966	8.27%	352	9.15%
Computerised CBT	1,904	1.98%	15	0.39%
Behavioural Activation (LI)	1,904	1.98%	67	1.74%
Other LI intervention	5,020	5.21%	206	5.35%
Psychoeducational peer-support	3,385	3.51%	158	4.10%
Other HI intervention	2,387	2.48%	98	2.55%
Behavioural Activation (HI)	484	0.50%	11	0.29%
Counseling	6,705	6.96%	608	15.80%
CBT	44,222	45.91%	1,263	32.81%
IPT	264	0.27%	17	0.44%
Missing	333	0.35%	11	0.29%
Total	96,330		3,849	

Table H4. Results of multinomial regression models

	**Relative Risk Ratios for 'Older Adult' (95% CIs)***
**LI pathway**	
Self-Help (book)§	0.93 (0.82-1.06)
Computerised CBT§	0.15 (0.09-0.25)
Behavioural Activation (LI)§	0.91 (0.70-1.18)
Other LI intervention§	0.86 (0.73-1.00)
Psychoeducational peer-support§	1.00 (0.84-1.19)
**HI pathway**	
Other HI intervention‡	1.27 (1.02-1.58)
Behavioural Activation (HI)‡	0.80 (0.43-1.48)
Counseling‡	2.58 (2.31-2.88)
IPT‡	2.20 (1.32-3.68)

Notes: * Adjusted for all clinical and sociodemographic variables; § reference category is "Guided Self-Help"; ‡ reference category is "CBT"

References.

Chambless, D.L., Caputo, G.C., Jasin, S.E., Gracely, E.J., Williams, C., 1985. The Mobility Inventory for Agoraphobia. Behav. Res. Ther. 23, 35–44. https://doi.org/10.1016/0005-7967(85)90140-8

Connor, K.M., Davidson, J.R.T., Churchill, L.E., Sherwood, A., Weisler, R.H., Foa, E., 2000. Psychometric properties of the Social Phobia Inventory (SPIN). Br. J. Psychiatry 176, 379–386. https://doi.org/10.1192/bjp.176.4.379

Creamer, M., Bell, R., Failla, S., 2003. Psychometric properties of the Impact of Event Scale - Revised. Behav. Res. Ther. 41, 1489–1496. https://doi.org/10.1016/j.brat.2003.07.010

Foa, E.B., Kozak, M.J., Salkovskis, P.M., Coles, M.E., Amir, N., 1998. The validation of a new obsessive-compulsive disorder scale: The obsessive-compulsive inventory. Psychol. Assess. 10, 206–214. https://doi.org/10.1037/1040-3590.10.3.206

Leuven, E., Sianesi, B., 2003. PSMATCH2: Stata module to perform full Mahalanobis and Propensity Score Matching, version 1.2. 1. Stat. Softw. Components.

NHS Digital, 2016. Psychological Therapies, Annual Report on the use of IAPT services: England 2015-16.

Salkovskis, P.M., Rimes, K.A., Warwick, H.M.C., Clark, D., 2002. The Health Anxiety Inventory: development and validation of scales for the measurement of health anxiety and hypochondriasis. Psychol. Med. 32, 843–53.

Shear, M.K., Rucci, P., Williams, J., Frank, E., Grochocinski, V., Vander Bilt, J., Houck, P., Wang, T., 2001. Reliability and validity of the Panic Disorder Severity Scale: Replication and extension. J. Psychiatr. Res. 35, 293–296. https://doi.org/10.1016/S0022-3956(01)00028-0

StataCorp, 2017. Stata Statistical Software: Release 15. 2017. https://doi.org/10.2307/2234838
